# Development of a respiratory severity score for hospitalized adults in a high HIV-prevalence setting—South Africa, 2010–2011

**DOI:** 10.1186/s12890-017-0368-8

**Published:** 2017-02-02

**Authors:** Alexander J. Millman, Adena Greenbaum, Sibongile Walaza, Adam L. Cohen, Michelle J. Groome, Carrie Reed, Meredith McMorrow, Stefano Tempia, Marietjie Venter, Florette K. Treurnicht, Shabir A. Madhi, Cheryl Cohen, Ebrahim Variava

**Affiliations:** 1 Influenza Division, Centers for Disease Control and Prevention, Atlanta, GA 30329 USA; 2 Epidemic Intelligence Service, Centers for Disease Control and Prevention, Atlanta, GA USA; 3 Influenza Program, Centers for Disease Control and Prevention, Pretoria, South Africa; 4 Centre for Respiratory Diseases and Meningitis, National Institute for Communicable Diseases of the National Health Laboratory Service, Johannesburg, South Africa; 5 School of Public Health, Faculty of Health Sciences, University of the Witwatersrand, Johannesburg, South Africa; 6 Medical Research Council, Respiratory and Meningeal Pathogens Research Unit, University of the Witwatersrand, Johannesburg, South Africa; 7 Global Disease Detection Center, Division of Global Health Protection, Centers for Disease Control and Prevention, Pretoria, South Africa; 8 Zoonoses Research Program, Department of Medical Virology, University of Pretoria, Pretoria, South Africa; 9 Department of Science and Technology/National Research Foundation: Vaccine Preventable Diseases, University of the Witwatersrand, Johannesburg, South Africa; 10 Department of Medicine, Klerksdorp-Tshepong Hospital Complex, Klerksdorp, South Africa; 11 Department of Medicine, Faculty of Health Sciences, University of the Witwatersrand, Johannesburg, South Africa; 12 Perinatal HIV Research Unit, University of the Witwatersrand, Johannesburg, South Africa

## Abstract

**Background:**

Acute lower respiratory tract infections (LRTI) are a frequent cause of hospitalization and mortality in South Africa; however, existing respiratory severity scores may underestimate mortality risk in HIV-infected adults in resource limited settings. A simple predictive clinical score for low-resource settings could aid healthcare providers in the management of patients hospitalized with LRTI.

**Methods:**

We analyzed 1,356 LRTI hospitalizations in adults aged ≥18 years enrolled in Severe Acute Respiratory Illness (SARI) surveillance in three South African hospitals from January 2010 to December 2011. Using demographic and clinical data at admission, we evaluated potential risk factors for in-hospital mortality. We evaluated three existing respiratory severity scores, CURB-65, CRB-65, and Classification Tree Analysis (CTA) Score assessing for discrimination and calibration. We then developed a new respiratory severity score using a multivariable logistic regression model for in-hospital mortality and assigned points to risk factors based on the coefficients in the multivariable model. Finally we evaluated the model statistically using bootstrap resampling techniques.

**Results:**

Of the 1,356 patients hospitalized with LRTI, 101 (7.4%) died while hospitalized. The CURB-65, CRB-65, and CTA scores had poor calibration and demonstrated low discrimination with c-statistics of 0.594, 0.548, and 0.569 respectively. Significant risk factors for in-hospital mortality included age ≥ 45 years (A), confusion on admission (C), HIV-infection (H), and serum blood urea nitrogen >7 mmol/L (U), which were used to create the seven-point ACHU clinical predictor score. In-hospital mortality, stratified by ACHU score was: score ≤1, 2.4%, score 2, 6.4%, score 3, 11.9%, and score ≥ 4, 29.3%. Final models showed good discrimination (c-statistic 0.789) and calibration (chi-square 1.6, Hosmer-Lemeshow goodness-of-fit *p*-value = 0.904) and discriminated well in the bootstrap sample (average optimism of 0.003).

**Conclusions:**

Existing clinical predictive scores underestimated mortality in a low resource setting with a high HIV burden. The ACHU score incorporates a simple set a risk factors that can accurately stratify patients ≥18 years of age with LRTI by in-hospital mortality risk. This score can quantify in-hospital mortality risk in an HIV-endemic, resource-limited setting with limited clinical information and if used to facilitate timely treatment may improve clinical outcomes.

**Electronic supplementary material:**

The online version of this article (doi:10.1186/s12890-017-0368-8) contains supplementary material, which is available to authorized users.

## Background

Acute lower respiratory tract infections (LRTI) are one of the most frequent causes of hospitalization in South Africa with an overall incidence ranging from 325 to 617/100,000 population annually [1]. In South Africa, HIV-infected individuals have higher rates of hospitalization and an increased risk of death from LRTI compared with HIV-uninfected individuals [1]. Many respiratory severity scores that use clinical and laboratory data have been developed to risk stratify and predict outcomes of patients hospitalized with pneumonia including the Pneumonia Severity Index (PSI) and CURB-65 scores [2–5]. Although these scores are used frequently in clinical practice, including in South Africa, they were developed and validated among HIV-uninfected adults in high income settings and may therefore underestimate mortality risk in HIV-infected adults in resource-limited settings [6]. Several respiratory severity scores have been developed for hospitalized HIV-infected adults and children [7–9], but they are not widely used or have not been prospectively validated to date. Having an accurate predictive respiratory severity score for low-resource settings could help healthcare providers and health systems better allocate limited resources and improve care by identifying patients at risk for severe outcomes.

We used data from Severe Acute Respiratory Illness (SARI) surveillance in South Africa, an active, prospective hospital-based surveillance system, to evaluate existing respiratory severity scores in a patient population with high HIV prevalence. We then sought to develop a novel severity score for LRTI in adults for use in resource-limited settings, including an assessment of its ability to predict mortality by evaluating the performance of the scoring model.

## Methods

### Ethics statement

The protocol was approved by the Research Ethics Committees of the University of the Witwatersrand (reference number M081042). This investigation was deemed non-research by the U.S. Centers for Disease Control and Prevention and did not need human subjects review by that institution. Written informed consent was obtained from adults aged ≥18 years upon enrollment in the SARI surveillance system for the collection of nasopharyngeal and throat swabs and blood specimens. All specimens collected from consenting patients were part of SARI surveillance or routine clinical care.

### SARI Surveillance

We performed a secondary analysis of data from hospitalized adults aged ≥18 years enrolled in SARI surveillance in South Africa from January 2010 through December 2011. SARI surveillance in South Africa has been previously described [10]. SARI was an active, prospective hospital-based surveillance program for cases of acute LRTI, defined as a hospitalized case in persons ≥5 years of age who met a modified 2011 World Health Organization (WHO) case definition for severe acute respiratory infection: sudden onset of fever (>38 °C) or reported fever, cough or sore throat, and shortness of breath or difficulty breathing with illness onset within 7 days prior to admission [11]. Surveillance officers review patient admission logs daily, consent, and enroll eligible cases, perform a medical chart review and patient interview and collect respiratory specimens for bacterial and viral pathogen testing. We included patients from three study sites in two provinces: (1) Tshepong Hospital, a public, semi-rural 300 bed hospital in Klerksdorp, North West Province; (2) Chris Hani Baragwanath Academic Hospital (CHBAH), a public, urban 3,200 bed hospital in Soweto, Gauteng Province; and (3) Selby Hospital, a private, urban 642 bed hospital in Johannesburg, Gauteng Province, that receives referrals of stable patients from area hospitals, including CHBAH. All hospitals have general medical wards and intensive care units (ICUs) with the capability for mechanical ventilation.

### Study population and data collection

The primary outcome was in-hospital mortality among patients ≥18 years of age hospitalized with LRTI. From January 2010 to December 2011, there were 1,988 SARI patients enrolled at Selby hospital, 991 SARI patients enrolled at CHBAH, and 76 SARI patients enrolled from Tshepong hospital. The study was powered for 10 degrees of freedom, which required 100 occurrences of death. In order to reach this sample size, we randomly sampled 60% of the SARI patients from Selby, CHBAH, and Tshepong hospital irrespective of outcome for chart review from the 2-year study period. This was estimated to provide 140 deaths.

We collected the following data for each enrolled SARI patient: demographics, underlying medical conditions, vital signs, signs and clinical examination findings, HIV status, and laboratory values including serum blood urea nitrogen (BUN), creatinine, and sodium. SARI surveillance records and the clinical laboratory database were the primary data sources, though investigators also performed retrospective chart review using a standardized data collection instrument to abstract data on several variables including mental status, blood pressure, and oxygen saturation which were not included in routine SARI data collection. We used the first available vital signs and laboratory values from the hospital admission as predictors for those variables. First available was defined as preferentially using vital signs and laboratory values recorded from the initial encounter in the casualty ward (emergency department); however, if values were unavailable from the casualty ward, we used the initial vital signs and laboratory values from the inpatient ward. Variables with >25% missing values were excluded from the analysis (Additional file 1).

### Assessment of risk factors for in-hospital death

To better understand important mortality predictors in this population, we evaluated the relationship between patient clinical characteristics and the primary outcome. We performed chi-square or Fisher’s exact tests to compare proportions for binary variables and the Wilcoxon rank sum test to evaluate distribution differences between continuous variables. All comparisons were 2-tailed and *p* < 0.05 was considered significant. We calculated unadjusted odds ratios and 95% confidence intervals (CI) for mortality using logistic regression.

### Evaluation of existing respiratory severity scores

We evaluated three existing respiratory severity scores, CURB-65, CRB-65, and Classification Tree Analysis (CTA) Score [3, 7], for our study population (Additional file 2: Table S1). We calculated the respective scores for each patient and the observed mortality risk by score category. We then evaluated the predictive accuracy of the scores by assessing the discrimination and calibration of each score for mortality in our study population (Additional file 2: Table S2) [3, 7]. Discrimination refers to the model’s ability to distinguish between individuals with and without the outcome of interest (in this case mortality) and was measured using the c-statistic or area under the receiver operating characteristic (ROC) curve [12]. Calibration measures the concordance of predicted and observed outcomes and was assessed visually as well as by using the Hosmer-Lemeshow goodness of fit test. [12] For the purposes of evaluating each score, we excluded individuals who had missing values for any of the predictor variables included in that severity score.

### Development of a new scoring system

To develop a new scoring system, we implemented a risk factor analysis for mortality by creating a multivariable logistic regression model using risk factors associated with mortality as identified in the univariate analysis with *p* < 0.2. We evaluated all included variables for interaction with one another, and then performed manual backward elimination in which non-significant variables were removed from the model one at a time starting with the variable with the smallest magnitude of effect until either all remaining variables had *p* < 0.05 or removing an additional variable significantly affected the model’s Akaike information criterion (AIC). The final multivariable logistic regression model was used to create a point-based scoring system in which “point values” were assigned by rounding the beta coefficient of each predictor in the model to the nearest integer [9].

### Internal validation

We evaluated the predictive accuracy of the new scoring system by assessing the discrimination and calibration of the model in our data. Since prediction models generally perform better on the initially observed population compared to new populations, we assessed this “optimism” by using standard bootstrap procedures [13]. To assess the internal validity of our model, bootstrap samples were drawn with replacement and of the same size as the original sample. The multivariable model was re-estimated in 300 bootstrap samples and each model was evaluated on the new sample and the original data. The average difference in the c-statistic indicates the optimism in the initially estimated discrimination [14].

All statistical analyses were performed using SAS version 9.3 (SAS Institute, Cary, NC).

## Results

### Study population and risk factors associated with mortality

From January 2010 to December 2011, there were 3,029 SARI cases including 218 (7.2%) deaths from CHBAH and Selby Hospital and 71 cases including 5 deaths from Tshepong Hospital. For our study, we evaluated the records of 1356 randomly selected SARI patients including 101 (7.4%) who died while hospitalized. Nine hundred forty-three (69.5%) patients were from Selby Hospital, 374 (27.6%) were from CHBAH, and 39 (2.9%) were from Tshepong Hospital. 1019/1325 (76.9%) were HIV-infected. Of the 376 (36.9%) HIV-infected patients with documented CD4 counts, the median CD4 count was 104 cells/mm^3^ (IQR 34.5–260.0 cells/mm^3^). Of the 1008 (98.9%) HIV-infected patients with documentation regarding antiretroviral therapy, 217 (21.5%) were known to be on treatment. 189/1356 (13.9%) of SARI patients included in this analysis had tuberculosis.

Chest radiography was performed on 843 (62.2%) of hospitalized LRTI patients. Pulse oximetry was documented in 745 (54.9%) patients, and 235 (17.3%) patients had documented receipt of any form of supplemental oxygen. No patients were admitted to the intensive care unit or were mechanically ventilated.

Table 1 shows risk factors associated with mortality for patients hospitalized with LRTI from univariate and multivariate analyses. Additionally, of the 745 patients with documented pulse oximetry, those who died had a lower oxygen saturation (median 92.5% (IQR 88–97%)) compared to those who were discharged (median 95.0% (IQR 91–97%) *p* = 0.02). Of the 96 individuals who received supplemental oxygen and had documented pulse oximetry, 49 (51.0%) had hypoxemia with an oxygen saturation less than 90%. Patients who died were more likely to have tuberculosis compared to those who were discharged (23/101 (22.8%) versus 166/1255 (13.2%), *p* = 0.01). Patients who died were more likely to have a longer delay between symptom onset and hospital admission than those who were discharged (median 5 days, (IQR 4–7) versus median 5 days (IQR 3–6), *p* = 0.02).

**Table 1:** Risk factors for inpatient-mortality among adults hospitalized with lower respiratory tract infection, South Africa, 2010–2011 (*N* = 1356)

Risk factor	Cases with available data (*N* = 1356)	Died *N* = 101, No. (%)	Discharged *N* = 1255, No. (%)	*p*-value^*^	Unadjusted odds ratio (95% confidence interval)	Adjusted odds ratio (95% confidence interval)
Age ≥ 45 years^†^	1356	67 (66.3)	561 (44.7)	<0.001	2.4 (1.6–3.7)	2.5 (1.4–4.2)
Male	1356	49 (48.5)	464 (37.0)	0.0214	1.6 (1.1–2.4)	0.8 (0.5–1.3)
Medical condition						
HIV	1325	87/98 (88.8)	932/1227 (76.0)	0.0038	2.5 (1.3–4.8)	2.9 (1.2–6.9)
Asthma	1356	2 (2)	82 (6.5)	0.0678	0.3 (0.1–1.2)	−
Diabetes	1356	3 (3)	43 (3.4)	1.0	0.9 (0.3–2.8)	−
Cardiovascular^§^	1356	2 (2)	41 (3.2)	0.77	0.6 (0.1–2.5)	−
Emphysema	1356	1 (1)	22 (1.8)	1.0	0.6 (0.1–4.2)	−
Cancer	1356	0 (0)	10 (0.8)	1.0	Not applicable	−
Other¶	1356	0 (0)	18 (1.4)	0.39	Not applicable	−
Clinical Data						
Confusion**	1356	14 (13.9)	24 (1.9)	<0.001	8.3 (4.1–16.5)	7.6 (3.0–19.5)
Received any supplemental oxygen	1356	35 (34.7)	200 (15.9)	<0.001	2.8 (1.8–4.3)	2.0 (1.2–3.5)
Temperature ≥38 °C	1328	20/99 (20.2)	299 (24.3)	0.355	0.8 (0.4–1.3)	−
Respiratory Rate >20 breaths per minute	1333	36/100 (36.0)	462/1233 (37.5)	0.770	0.9 (0.6–1.4)	−
Heart Rate ≥115 beats per minute	1347	50/100 (50.0)	493/1247 (39.5)	0.040	1.5 (1.0–2.3)	1.6 (0.9–2.7)
Hypotension (Systolic <90 mmHg or diastolic ≤60 mmHg)	1352	24/101 (23.8)	249/1251 (19.9)	0.353	1.3 (0.8–2.0)	−
Serum Sodium <135 mmol/L	1029	54/80 (67.5)	478/949 (50.4)	0.003	2.0 (1.3–3.3)	1.6 (0.9–2.8)
Urea >7 mmol/L	1029	55/80 (68.8)	274/949 (28.9)	<0.001	5.4 (3.3–8.9)	4.4 (2.5–7.6)
Serum Creatinine ≥ 106.08 μmol/L	1028	34/80 (42.5)	190/948 (20.0)	<0.001	2.9 (1.8–4.7)	−^††^

^*^ *p*-value refers to chi-square or Fischer’s exact test for categorical variables and to a Wilcoxon rank sum test for continuous variables

^†^Age (median, interquartile range, years) Died 45 (35–53) versus Discharged 38 (30–48) *p* >0.001

^§^Includes cardiac and cerebrovascular disease

^¶^Other includes liver disease, renal disease, spinal disorders, obesity, pregnancy, chemotherapy, autoimmune diseases, and seizure disorders

^**^“Confusion” based on positive documentation in the medical chart. A no or lack of documentation in the medical chart was classified as “not confused”

^††^Not included in final multivariate analysis because it demonstrated co-linearity with urea

### Evaluation of existing severity scores in a high HIV prevalence setting

Table 2 shows predicted and observed mortality risk based on the risk classification by CURB-65, CRB-65 and the CTA scores. The majority of our patient population received the lowest possible score in each of the existing scoring systems including the majority of those with in-hospital death. For example, 848 (83.9%) patients were classified as low-risk using CURB-65, equivalent to a CURB-65 score of 0 or 1 that correlates to a 1.5% predicted risk of 30-day mortality. However, in our population, we observed 6.4% in-hospital mortality for individuals classified as low-risk using CURB-65. These existing scores demonstrated low discrimination with c-statistics of 0.594, 0.548, and 0.569 for the CURB-65, CRB-65, and CTA scores respectively. Each of the scores also demonstrated poor calibration. In particular for the CURB-65 and CRB-65 scores, the majority of individuals were unable to attain the highest possible score because they did not meet the respiratory, blood pressure or age criteria of these scores.

**Table 2:** Predicted and observed risk of mortality based on risk classification by CURB-65, CRB-65, CTA, and CURB-45 severity scores among hospitalized adults with lower respiratory tract infections, South Africa, 2010–2011

Severity Score Risk Classification	Score	N	Predicted Mortality (%)	Observed Mortality (%) (95% Confidence Interval)	C-statistic
CURB-65 (*N* = 1011)					
Low risk	0 or 1	848	1.5	6.4 (4.8–8.2)	0.594
Intermediate risk	2	140	9.2	12.7 (7.8–19.6)	
High risk	≥3	23	22.0	34.8 (15.3–54.3)	
CRB-65 (*N* = 1332)					
Low risk	0	905	1.2	6.5 (5.0–8.3)	0.548
Intermediate risk	1 or 2	427	8.2	9.6 (7.0–12.8)	
High risk	3 or 4	0	31.0	0	
CTA (*N* = 1011)					
Stage 1	−	815	2.3	6.9 (5.2–8.8)	0.569
Stage 2	−	114	5.8	7.0 (3.1–13.4)	
Stage 3	−	25	12.9	36.0 (18.0–57.5)	
Stage 4	−	52	22.0	7.7 (2.1–18.5)	
Stage 5	−	5	40.5	60.0 (14.7–94.7)	
CURB-45 (*N* = 1011)					
Low risk	0 or 1	751	−	4.9 (3.5–6.7)	0.666
Intermediate risk	2	204	−	12.8 (8.5–18.1)	
High risk	≥3	56	−	21.3 (18.8–44.1)	

Legend: The CURB-65 and CRB-65 scores predict 30-day mortality risk. The Classification Tree Analysis predicts inpatient mortality risk for HIV-infected patients

Given the younger median age in our patient cohort, we modified the CURB-65 score to assign one point for an age ≥ 45 years (henceforth CURB-45), to see if the predictive accuracy of the score would improve. Forty-five years of age was selected as the cut off since it was the median age of the individuals who died. Table 2 shows the predicted and observed risk of mortality for the CURB-45 score. The modified score demonstrated improved discrimination compared to CURB-65 (c-statistic 0.666 versus 0.594). Additionally, the CURB-45 score had improved calibration compared to the CURB-65 score.

### Development of a new respiratory severity score

Since the existing severity scores underestimated mortality risk in our population, we developed a multivariable logistic regression model for in-hospital mortality using identified risk factors associated with mortality in our study population. These risk factors included age ≥ 45 years, sex, HIV status, heart rate ≥ 115 beats per minute, the presence of confusion on clinical examination, hyponatremia, (serum sodium <135 mEq/L), elevated urea (serum BUN > 7 mmol/L), and receipt of supplemental oxygen. Serum creatinine and BUN demonstrated co-linearity, so we only evaluated serum BUN in the final model since it has been applied to other commonly used severity scores. Using a manual backward elimination method, sex, heart rate, and serum sodium were removed from all final models. Although receipt of supplemental oxygen was significant, the removal of this variable resulted in a more parsimonious model without significantly affecting the AIC. Therefore, the final model included, age (A), confusion (C), HIV status (H) and serum BUN (U for uremia) which we will now refer to as the ACHU score.

Points (e.g. rounding of the beta coefficient to the nearest integer) assigned to each of the risk factors in the final model are shown in Table 3. An overall score was calculated for each patient for an episode of LRTI by adding the points for each identified risk factor. The new severity score ranged from 0 to 6, and we stratified the score as follows: a score of ≤1 was assigned a risk classification of low; a score of 2 was assigned a risk classification of low-intermediate; a score of 3 was assigned a risk classification of high-intermediate; and score ≥ 4 was assigned a risk classification of high (Table 4 and Additional file 2: Table S3). The median risk classification was low-intermediate. For each risk classification, the observed risk of mortality and predicted probability of death from the model is shown in Table 4.

**Table 3:** Risk factors for inpatient mortality from the final multivariable model for adults hospitalized with LRTI with ACHU (age, confusion, HIV, urea) respiratory severity score, South Africa, 2010–2011

Risk factor	Adjusted Odds Ratio (95% Confidence Interval)	*p*-value	Beta (unexponentiated regression coefficient)	Score^a^
Age ≥ 45	2.3 (1.4–3.9)	0.001	0.85	1
Confusion	9.3 (3.8–22.4)	<0.001	2.23	2
HIV-infected	4.2 (1.8–10.1)	0.001	1.44	1
Urea > 7 mmol/L	4.6 (2.7–7.8)	<0.001	1.53	2

^a^The point value assigned for the score was determined by rounding the beta coefficient to the nearest integer

**Table 4:** Predicted and observed risk of mortality based on risk classification using the ACHU (Age, confusion, HIV, urea) respiratory severity score among hospitalized adults with lower respiratory tract infections, South Africa, 2010–2011

ACHU Risk Classification^a^	Score	N	Predicted Mortality (%)	Observed Mortality (%) (95% Confidence Interval)	C-statistic
Low	≤1	570	2.4	2.1 (0.1–3.7)	0.769
Low-intermediate	2	110	6.4	6.4 (2.6–12.7)	
High-intermediate	3	230	11.9	14.4 (10.1–19.6)	
High	≥4	101	29.3	25.7 (17.6–35.4)	

^a^ACHU respiratory severity score risk classification categories are based on the number of points an individual patient has based on the presence or absence of risk factors associated with in-hospital mortality from our population. The ACHU scores range from 0 to 6

### Validation of the ACHU score

We measured the discrimination and calibration to evaluate the performance of the ACHU score. In the full dataset, models using the risk classifications showed good discrimination (c-statistic 0.789) and calibration (chi-square 1.6, Hosmer-Lemeshow goodness-of-fit *p*-value = 0.904). To assess the internal validity of our model, we used bootstrapping techniques to estimate the optimism in our measurement of model discrimination. From 300 bootstrap samples, the c-statistic ranged from 0.764 to 0.792 with an estimated average optimism of 0.003, which indicates that the model discriminates well when applied to the bootstrapped sample.

## Discussion

South African adults hospitalized with LRTI had a high HIV prevalence and an in-hospital mortality rate of 7.4%. Existing and modified respiratory severity scores were poor predictors of mortality and underestimated in-hospital mortality in our population. We developed and validated a novel clinical prediction score which uses a simple and commonly available set of clinical factors and laboratory tests to discriminate between adults with varying in-hospital mortality risk from LRTI. A combination of four factors (age ≥ 45 years, confusion on clinical exam, HIV-infection and BUN >7 mmol/L) in a scoring model provide good discrimination and calibration for predicting in-hospital mortality in adults with LRTI and could be used by clinicians in resource-limited settings with high HIV prevalence to identify pneumonia patients with elevated risk of death.

This simplified score combines elements of existing pneumonia severity scores and has the ability to be applied to HIV-infected and HIV-uninfected individuals. We believe that our use of existing surveillance data from three South African hospitals accurately reflects the variability of “real world” clinical information available to clinical staff caring for patients hospitalized with LRTI. Some factors included in other scores were not appropriate for our novel score. For example, chest X-rays were only performed on ~60% of individuals. In addition, while there was a significant difference between receipt of supplemental oxygen among deaths and discharges, we found that among those with a documented oxygen saturation, receipt of supplemental oxygen correlated poorly with documented hypoxemia thus limiting its reliability as a surrogate marker for hypoxemia.

Our in-hospital mortality rate of 7.4% likely reflects the high burden of HIV and the high frequency of pulmonary tuberculosis in our study population—a subset of LRTI cases identified in South Africa’s SARI surveillance system. Our population appears to be similar to the overall population of LRTI cases in the SARI surveillance system, which reported an overall case-fatality ratio of 7.2% [1]. Additionally, the prevalence of HIV (76.8%) and median age of death among the subgroup in our analysis was comparable to those in the overall SARI population [1]. In South Africa, HIV prevalence was estimated to be 17% in individuals 15–49 years of age in 2012; [15] however, our findings illustrate the high burden of co-existing HIV among patients hospitalized with LRTI in South Africa. This problem has been previously described, and the incidence of LRTI hospitalizations for HIV-infected individuals has been shown to be higher than what has been described from low HIV-prevalence middle income countries such as Bangladesh (prevalence rate estimate from 2013: 0.1%) and other high HIV-prevalence African countries such as Kenya (prevalence rate estimate from 2013: 6.0%) [1].

Existing severity scores such as CURB-65 performed poorly in our study population because they underestimated the risk of death. One of the main limitations of these severity scores is that they excluded HIV-infected individuals [3]. However, we also found that Arozullah’s CTA score, which had been validated in an HIV-infected population also had poor discrimination and calibration and generally underestimated mortality in our study population [7]. Our study population was generally unable to attain higher risk classifications in these scores because they did not meet that age threshold (CURB-65 and CRB-65) or the blood pressure or respiratory rate thresholds (CURB-65, CRB-65, and CTA). We were unable to evaluate other existing severity scores such as PSI, PIRO, and SMART-COP because required laboratory and clinical findings were not commonly gathered in our setting; thus, a new score was needed for use with commonly available data [2, 4, 5]. However, some of these scores would also be inappropriate for resource limited-settings, such as ours in which availability of intensive care units and mechanical ventilator may not be comparable to high income country settings. We did find improved discrimination when we modified the CURB-65 score to have an age cut-off of 45 years of age; however, it is still based on a predictive model that excluded HIV-infected individuals.

A clinical predictive score from Uganda for 30-day mortality in HIV-infected adults was recently developed. Their study population of 835 patients was similar to ours in terms of HIV status, young age, low antiretroviral coverage, and high frequency of pulmonary tuberculosis [8]. Koss et al. identified four clinical predictors (heart rate above 120 beats/min, respiratory rate above 30 breaths/min, oxygen saturation below 90%, and CD4 cell count below 50 cells/mm^3^), which were predictive of increased risk of 30-day mortality [8]. The value of these predictors have been identified before in other severity scores in adults and children [2, 3, 7, 9]. We were unable to evaluate the predictive accuracy of their score on our study population because we had CD4 cell count information or oxygen saturation values recorded for only a limited subset of our study population. Some of these criteria such as measurement of oxygen saturation may not be widely available in resource-limited settings, which would limit the ability to use their score appropriately. Unlike in their study, we did not find heart rate or respiratory rate to be predictive of increased risk of mortality. This differs from other studies which have identified these as risk factors particularly respiratory rate [3, 7, 9]. Finally, Koss et al. found a 30-day mortality rate of 18.2% among patients hospitalized with pneumonia in a similar patient population of Ugandan adults [8]. The higher mortality rate found in Koss et al. may be attributable to differences in quality of care and resources in South African hospitals compared to Ugandan hospitals as well as to patients who died after hospital discharged—a period we did not measure in our study.

Our results are subject to several limitations. First, our mortality outcome was limited to in-hospital mortality since SARI surveillance does not prospectively follow patients after hospital discharge; therefore, unlike with other severity scores such as CURB-65, which predict 30-day mortality risk, we were unable to evaluate our score’s predictive accuracy for deaths that occur in the post-discharge period. Second, many patients were missing data on various risk factors such as oxygen saturation or routine laboratory tests, which have been shown to be mortality risk factors in other severity scores, and were therefore unable to account for them in our model. While this is a limitation, our score was developed in a “real world” clinical setting and is therefore useful for settings in which clinical data such as oxygen saturation or specialized laboratory tests are not routinely available. Finally, since data on CD4 count and antiretroviral therapy were unavailable on the majority of HIV-infected patients, we were unable to evaluate for differences in severity of HIV status.

## Conclusion

Our ACHU score incorporates a simple set of predictor variables that predict the probability of in-hospital mortality in adults hospitalized with LRTI in a middle income country-setting with a high prevalence of HIV. The ability to quantify the risk of in-hospital mortality in a resource-limited setting with limited clinical information may improve the clinical outcomes of a vulnerable population by better informing triage decisions on admission. With further validation in other populations, this new respiratory severity score may provide middle income countries with a high HIV-prevalence with a tool to more effectively manage LRTI and to design clinical and public health interventions to reduce mortality based on the severity of LRTI in hospitalized adults.

## Additional files

Additional file 1: BMC Pulmonary_Severity Score Data.xlsx. Severity Score Dataset. Dataset generated and used for analysis and creation of the ACHU score. Two tabs are included 1) includes the data used for the analysis 2) includes important notes related to the analytical methods and definitions for several composite variables. (XLS 1235 kb)
Additional file 2: Table S1. CURB-65, CRB-65, Classification Tree Analysis (CTA) severity scores. **Table S2.** Predicted and observed risk of mortality based on CURB-65, CRB-65, Classification Tree Analysis (CTA), and CURB-45 severity scores among hospitalized adults with lower respiratory tract infections, South Africa, 2010–2011. **Table S3.** Predicted and observed risk of mortality based by ACHU (Age, confusion, HIV, urea) respiratory severity score among hospitalized adults with lower respiratory tract infections, South Africa, 2010–2011. (DOCX 21 kb)

## Abbreviations

BUN: Blood urea nitrogen
LRTI: Lower respiratory tract infection
SARI: Severe acute respiratory illness
