# Supplementary material for: Development of a respiratory severity score for hospitalized adults in a high HIV-prevalence setting—South Africa, 2010–2011
Source: BMC Pulm Med. 2017 Feb 2;17:28. doi: 10.1186/s12890-017-0368-8 (PMC5288997; doi:10.1186/s12890-017-0368-8)
Supplement: Additional file 2: Table S1. — CURB-65, CRB-65, Classification Tree Analysis (CTA) severity scores. Table S2. Predicted and observed risk of mortality based on CURB-65, CRB-65, Classification Tree Analysis (CTA), and CURB-45 severity scores among hospitalized adults with lower respiratory tract infections, South Africa, 2010–2011. Table S3. Predicted and observed risk of mortality based by ACHU (Age, confusion, HIV, urea) respiratory severity score among hospitalized adults with lower respiratory tract infections, South Africa, 2010–2011. (DOCX 21 kb) [file 12890_2017_368_MOESM2_ESM.docx]

**Supplementary Table 1:** CURB-65, CRB-65, Classification Tree Analysis (CTA) severity scores

| **Score** | **Predictors** | **Score** | **Risk Classification** | **Predicted morality, %** |
| --- | --- | --- | --- | --- |
| CURB-65 | Confusion  Urea >7 mmol/l  Respiratory Rate ≥30/min  Blood Pressure (SBP <90 mmHg or DBP ≤60mmHg)  Age ≥65 years | 0 | Low risk | 1.5 |
|  |  | 1 |  |  |
|  |  | 2 | Intermediate risk | 9.2 |
|  |  | 3 | High risk | 22.0 |
|  |  | 4 |  |  |
|  |  | 5 |  |  |
| CRB-65 | Confusion  Urea >7 mmol/l  Respiratory Rate ≥30/min  Blood Pressure (SBP <90 mmHg or DBP ≤60mmHg) | 0 | Low risk | 1.2 |
|  |  | 1 | Intermediate risk | 8.2 |
|  |  | 2 |  |  |
|  |  | 3 | High risk | 31.0 |
|  |  | 4 |  |  |
| CTA | Neurologic symptoms  Respiratory rate ≥ 25/min  Creatinine >1.2 mg/dl | 1 | Stage 1  (No symptoms) | 2.3 |
|  |  | 2 | Stage 2  (Respiratory rate) | 5.8 |
|  |  | 3 | Stage 3  (Neurologic symptoms) | 12.9 |
|  |  | 4 | Stage 4  (Respiratory rate and creatinine) | 22.0 |
|  |  | 5 | Stage 5  (Neurologic symptoms and respiratory rate) | 40.5 |

Legend: The CURB-65 and CRB-65 scores predict 30-day mortality risk. The Classification Tree Analysis predicts in-hospital mortality risk for HIV-infected patients.

**Supplementary Table 2**: Predicted and observed risk of mortality based on CURB-65, CRB-65, Classification Tree Analysis (CTA), and CURB-45 severity scores among hospitalized adults with lower respiratory tract infections, South Africa, 2010–2011

| CURB-65 Score (N=1011) | Predicted mortality (%) | N | Died | Discharged |
| --- | --- | --- | --- | --- |
| 0 | 0 | 497 | 15 (3.0) | 482 (97.0) |
| 1 | 0 | 351 | 39 (11.1) | 312 (88.9) |
| 2 | 8.3 | 140 | 18 (12.9) | 122 (87.1) |
| 3 | 21.4 | 23 | 8 (34.8) | 15 (65.2) |
| 4 | 26.3 | 0 | 0 (0) | 0 (0) |
| 5 | 33.3 | 0 | 0 (0) | 0 (0) |
| CRB-65 Score (N=1332) |  |  |  |  |
| 0 | 0 | 905 | 59 (6.5) | 846 (93.5) |
| 1 | 5.1 | 374 | 30 (8.0) | 344 (92.0) |
| 2 | 11.3 | 53 | 11 (20.8) | 42 (79.3) |
| 3 | 30.8 | 0 | 0 (0) | 0 (0) |
| 4 | 33.3 | 0 | 0 (0) | 0 (0) |
| CTA Stage (N=1011) |  |  |  |  |
| Stage 1 | 2.3 | 815 | 56 (6.9) | 759 (93.1) |
| Stage 2 | 5.8 | 114 | 8 (7.0) | 106 (93.0) |
| Stage 3 | 12.9 | 25 | 9 (36.0) | 16 (64.0) |
| Stage 4 | 22.0 | 52 | 4 (7.7) | 48 (92.3) |
| Stage 5 | 40.5 | 5 | 3 (60.0) | 2 (40.0) |
| CURB-45 Score (N=1011) |  |  |  |  |
| 0 | - | 361 | 9 (2.5) | 352 (97.5) |
| 1 | - | 390 | 28 (7.2) | 362 (92.8) |
| 2 | - | 204 | 26 (12.8) | 178 (87.3) |
| 3 | - | 47 | 13 (27.7) | 34 (72.3) |
| 4 | - | 9 | 4 (44.4) | 5 (55.6) |
| 5 | - | 0 | 0 | 0 |

Legend: The CURB-65 and CRB-65 scores predict 30-day mortality risk. The Classification Tree Analysis predicts in-hospital mortality risk for HIV-infected patients.

**Supplementary Table 3**: Predicted and observed risk of mortality based by ACHU (Age, confusion, HIV, urea) respiratory severity score among hospitalized adults with lower respiratory tract infections, South Africa, 2010–2011

| ACHU score (N=1011) | Predicted mortality (%) | N | Died | Discharged |
| --- | --- | --- | --- | --- |
| 0 | 0.1 | 84 | 0 (0) | 84 (100) |
| 1 | 2.7 | 486 | 12 (2.5) | 474 (97.5) |
| 2 | 6.4 | 110 | 7 (6.4) | 103 (93.6) |
| 3 | 11.9 | 230 | 33 (14.4) | 197 (85.7) |
| 4 | 25.0 | 88 | 20 (22.7) | 68 (77.3) |
| 5 | 51.8 | 9 | 2 (22.2) | 7 (77.8) |
| 6 | 75.3 | 4 | 4 (100) | 0 (0) |
